# Prognostics by Proxy

**Published:** 1898-09

**Authors:** 


### PROGNOSTICS BY PROXY.

It is related of a noted English bishop who had for years nursed the fear that he would some day become paralyzed, that on one occasion at a dinner, he suddenly interrupted the guests at the table by exclaiming that his worst fears had been realized at last; that he was paralyzed in his right lower limb; that he had been pinching his thighs for some moments and was unable to detect the slightest feeling. A lady sitting next to him assured him that he was mistaken, for it was her limb he had been pinching instead of his, the silk of the lady's dress being difficult to detect from the silk of the bishop's robe.

